# rMATS-cloud: Large-scale Alternative Splicing Analysis in the Cloud

**DOI:** 10.1093/gpbjnl/qzaf036

**Published:** 2025-04-26

**Authors:** Jenea I Adams, Eric Kutschera, Qiang Hu, Chun-Jie Liu, Qian Liu, Kathryn Kadash-Edmondson, Song Liu, Yi Xing

**Affiliations:** Genomics and Computational Biology Graduate Group, University of Pennsylvania, Philadelphia, PA 19104, USA; Center for Computational and Genomic Medicine, The Children’s Hospital of Philadelphia, Philadelphia, PA 19104, USA; Center for Computational and Genomic Medicine, The Children’s Hospital of Philadelphia, Philadelphia, PA 19104, USA; Department of Biostatistics and Bioinformatics, Roswell Park Comprehensive Cancer Center, Buffalo, NY 14203, USA; Center for Computational and Genomic Medicine, The Children’s Hospital of Philadelphia, Philadelphia, PA 19104, USA; Department of Biostatistics and Bioinformatics, Roswell Park Comprehensive Cancer Center, Buffalo, NY 14203, USA; Center for Computational and Genomic Medicine, The Children’s Hospital of Philadelphia, Philadelphia, PA 19104, USA; Department of Biostatistics and Bioinformatics, Roswell Park Comprehensive Cancer Center, Buffalo, NY 14203, USA; Center for Computational and Genomic Medicine, The Children’s Hospital of Philadelphia, Philadelphia, PA 19104, USA; Department of Pathology and Laboratory Medicine, University of Pennsylvania, Philadelphia, PA 19104, USA; Department of Biomedical and Health Informatics, The Children’s Hospital of Philadelphia, Philadelphia, PA 19104, USA

**Keywords:** Bioinformatics, Cloud computing, Transcriptomics, RNA-seq, Alternative splicing

## Abstract

Although gene expression analysis pipelines are often a standard part of bioinformatics analysis, with many publicly available cloud workflows, cloud-based alternative splicing analysis tools remain limited. Our lab released rMATS in 2014 and has continuously maintained it, providing a fast and versatile solution for quantifying alternative splicing from RNA sequencing (RNA-seq) data. Here, we present rMATS-cloud, a portable version of the rMATS workflow that can be run in virtually any cloud environment suited for biomedical research. We compared the time and cost of running rMATS-cloud with two RNA-seq datasets on three different platforms (Cavatica, Terra, and Seqera). Our findings demonstrate that rMATS-cloud handles RNA-seq datasets with thousands of samples, and therefore is ideally suited for the storage capacities of many cloud data repositories. rMATS-cloud is available at https://dockstore.org/workflows/github.com/Xinglab/rmats-turbo/rmats-turbo-cwl, https://dockstore.org/workflows/github.com/Xinglab/rmats-turbo/rmats-turbo-wdl, and https://dockstore.org/workflows/github.com/Xinglab/rmats-turbo/rmats-turbo-nextflow.

## Graphical abstract

**Figure:**
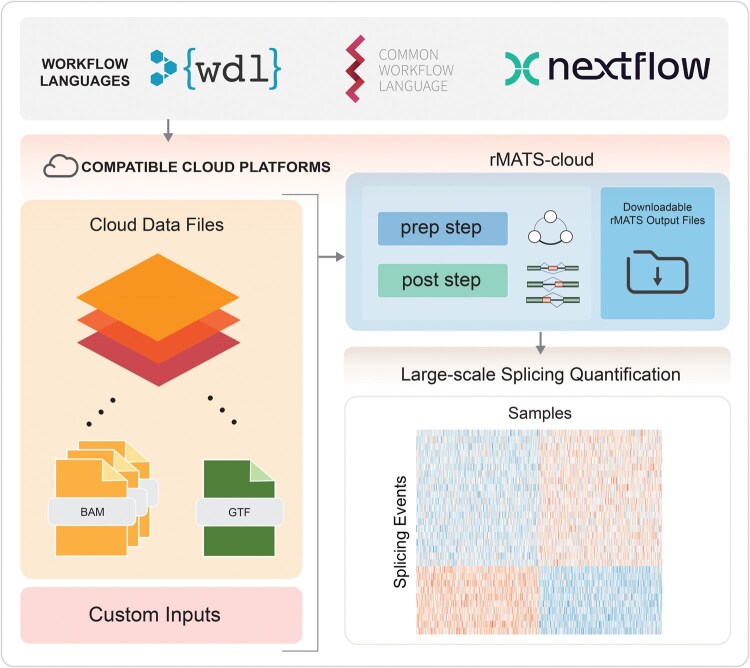

## Introduction

Today’s multi-omic landscape is fueling ever-growing high-dimensional data with expansive storage and analysis needs. In response, the biomedical research community is increasingly turning to cloud storage rather than housing data solely on private high-performance computing (HPC) systems. With the right tools, researchers can now run end-to-end analysis workflows in environments that also act as highly secure data repositories. Cloud storage sites may also house large-scale biomedical datasets with their built-in analysis suites. Ultimately, cloud computing is changing how researchers collaborate, work, and share reproducible research in ways that save money and time by eliminating the need to download and store massive datasets.

Cloud computing enables the storage and analysis of large-scale, multi-omic (genomic, transcriptomic, proteomic, metabolomic, *etc.*) data. With the prevalence of high-throughput sequencing technologies, RNA sequencing (RNA-seq) data have become a highly abundant data type for studying gene expression and RNA processing. One of the key steps in RNA processing is RNA splicing, which is the removal of intronic regions and the joining of exonic regions of precursor mRNA [1]. Moreover, alternative splicing can generate multiple transcript and protein isoforms from individual gene loci, greatly enhancing the regulatory and phenotypic diversity of eukaryotic organisms [2]. Using RNA-seq, researchers can quantify splicing events by counting the number of reads that map to individual splice junctions.

Although gene expression analysis pipelines are often a standard part of bioinformatics analysis, with many publicly available cloud workflows, cloud-based alternative splicing analysis tools remain limited. rMATS-turbo [3], which has been maintained by our lab since 2017 after its predecessor rMATS was published in 2014 [4], discovers and quantifies alternative splicing events from large-scale RNA-seq data. We recently developed rMATS-cloud, a portable version of the rMATS-turbo workflow that can be run in virtually any cloud environment suited for biomedical research. rMATS-cloud handles RNA-seq datasets with thousands of samples, making it ideally suited for the storage capacities of many cloud data repositories which already house large RNA-seq datasets.

## Implementation

rMATS-cloud currently supports three central workflow languages: Workflow Description Language (WDL) [5], Common Workflow Language (CWL) [6], and Nextflow [7] (**Table 1**). This flexibility allows users to integrate rMATS-cloud into existing workflows on various platforms, including Terra [5], CAVATICA (https://cavatica.sbgenomics.com/), Cancer Genomics Cloud [8], and more. These workflows are available on Dockstore [9] and GitHub (https://github.com/Xinglab/rmats-turbo/).

**Table 1:** Features of rMATS-cloud compatible platforms and workflows

Feature	CWL	WDL	Nextflow
Cloud platform	BioData Catalyst (https://biodatacatalyst.nhlbi.nih.gov/) Cancer Genomics Cloud (https://www.cancergenomicscloud.org/) Cavatica (https://cavatica.sbgenomics.com/)	AnVIL (https://anvilproject.org/) BioData Catalyst (https://biodatacatalyst.nhlbi.nih.gov/) DNAnexus (http://dnanexus.com/) Terra (https://anvil.terra.bio/)	Seqera (https://seqera.io/platform/)
URL	https://dockstore.org/workflows/github.com/Xinglab/rmats-turbo/rmats-turbo-cwl	https://dockstore.org/workflows/github.com/Xinglab/rmats-turbo/rmats-turbo-wdl	https://dockstore.org/workflows/github.com/Xinglab/rmats-turbo/rmats-turbo-nextflow

*Note*: Each column represents a workflow language, and the rows show compatible cloud platforms and rMATS-cloud implementations supporting that language. CWL, Common Workflow Language; WDL, Workflow Description Language.

Cloud environments that support these workflow languages include Cancer Genomics Cloud and CAVATICA for CWL, AnVIL, Biodata Catalyst, DNAnexus, and Terra for WDL, and Seqera for Nextflow. These platforms share key features such as scalability, reproducibility, containerized execution, and compatibility with HPC systems. The primary distinction between them lies in the workflow language each supports: CWL, WDL, or Nextflow. Among these, WDL is known for its relatively readable syntax, whereas CWL can be more complex to read. Nonetheless, the high portability of these workflow languages enables them to meet a wide array of computational needs.

**Figure 1:**
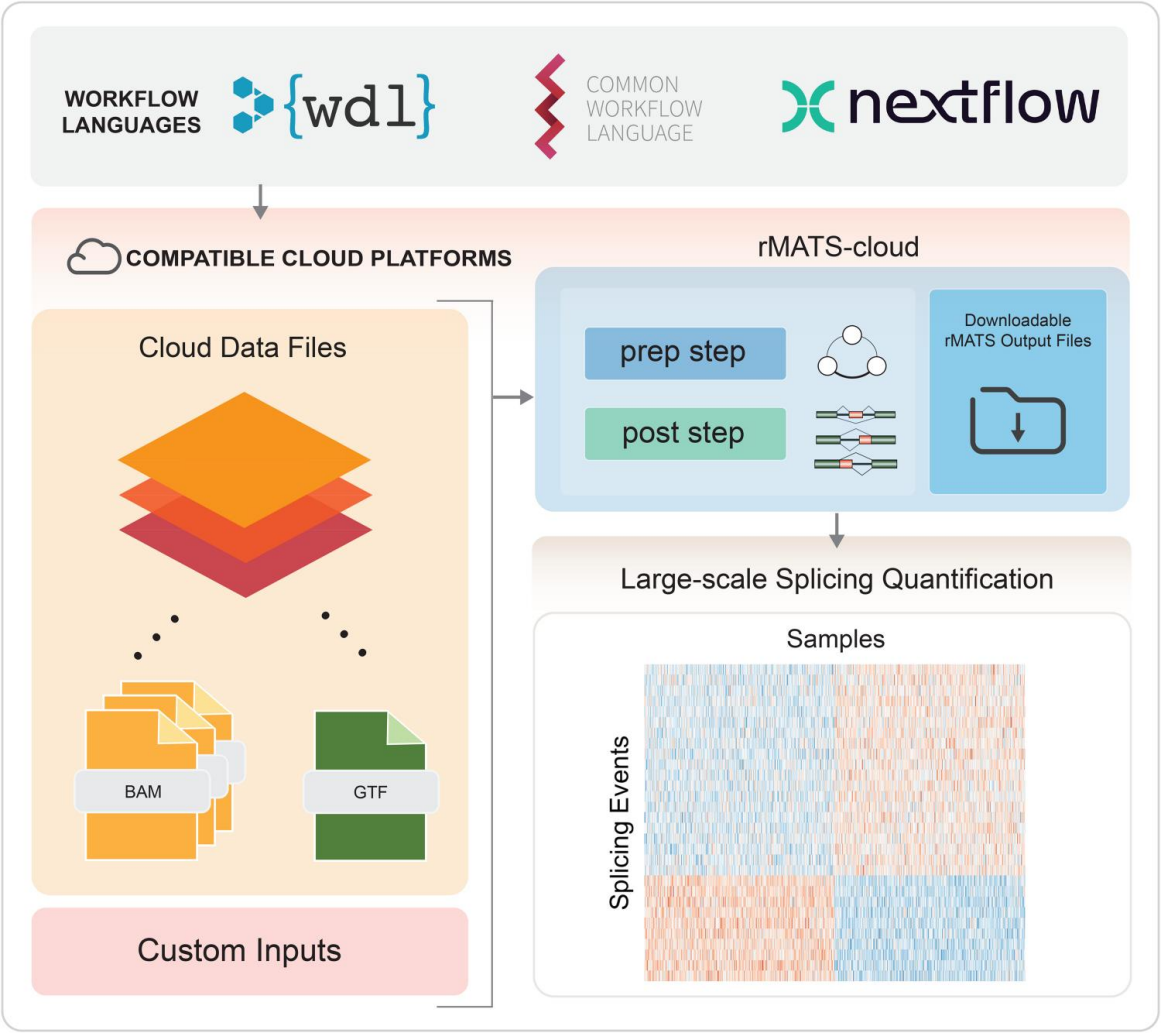
Schematic of rMATS-cloud workflow rMATS-cloud currently supports three main workflow management systems: WDL, CWL, and Nextflow. The rMATS-cloud workflow takes BAM files for RNA-seq data and a GTF file for gene and transcript annotations, in the form of cloud platform paths or a compiled data file. Next, the rMATS-turbo workflow runs end to end with its prep step and post step, yielding output files that can be downloaded to a local file system for downstream analysis. A mock dataset was used to generate the heatmap shown. WDL, Workflow Description Language; CWL, Common Workflow Language; RNA-seq, RNA sequencing.

As shown in **Figure 1**, rMATS-cloud starts with BAM files for each relevant RNA-seq sample or sample group, and a GTF file for gene and transcript annotations. Users can modify any of the input parameters through the workflow config files, including read length, novel splice site detection (on/off), and choice of statistical method. In the rMATS-cloud workflow, the rMATS-turbo prep step is run on each BAM file to compute splicing graphs for each gene. The prep step can be parallelized across machines in the cloud platform to utilize available resources. Once the prep steps are complete, the post step combines the splicing graphs from all of the prep steps and quantifies alternative splicing events by type. While downstream analysis can be done in the cloud, the output files are relatively small compared to the input BAM files, making it convenient to download them for local analysis and visualization if preferred.

## Results

We ran rMATS-cloud on (1) a small-scale RNA-seq dataset from 2 prostate cancer cell lines with contrasting epithelial *versus* mesenchymal phenotypes, each with three biological replicates [10] (EMT Dataset; SRA: PRJNA438990) and (2) a large-scale RNA-seq dataset of pediatric acute myeloid leukemia (AML) patient samples from the Children’s Oncology Group (COG Dataset, https://cavatica.sbgenomics.com/u/kids-first-drc/sd-pet7q6f2; AAML1031 clinical trial, NCT01371981-D4). We compared the time and cost of running rMATS-cloud on three different platforms (Cavatica, Terra, and Seqera). The results are shown in **Table 2**. To test the EMT Dataset, we ran three runs of the same workflow and averaged the cost and time of the runs. Six BAM files for the EMT Dataset were processed in 15.3–49.7 min for 5–25 cents. Because the COG Dataset is securely housed within the Cavatica platform, we only tested it on one platform with one run to cut costs. In Cavatica, rMATS-cloud processed 1113 BAM files in 5 h and 41 min for USD $38.70.

**Table 2:** rMATS-cloud enables large-scale alternative splicing analysis in the cloud

Platform (workflow language)	EMT Dataset (6 cell line samples; 249.2 million reads per BAM on average)		COG Dataset (1113 patient samples; 173.7 million reads per BAM on average)	
	Average time	Average cost	Time	Cost
Cavatica (CWL)	49.7 min	$0.25 (USD)	5 h 41 min	$38.70 (USD)
Terra (WDL)	42.3 min	$0.17 (USD)		
Seqera (Nextflow)	15.3 min	$0.05 (USD)		

*Note*: Time and cost of running rMATS-cloud are shown for the EMT and COG datasets. For the EMT Dataset, rMATS-cloud performance is shown across different cloud platforms supporting diverse workflow languages. This dataset was obtained from 2 prostate cancer cell lines with contrasting epithelial *versus* mesenchymal phenotypes, each with three biological replicates [10] (SRA: PRJNA438990). EMT, epithelial to mesenchymal transition; COG, Children’s Oncology Group.

## Discussion

Here, we present a solution for processing large-scale RNA-seq data for alternative splicing analysis in the cloud. rMATS-cloud is now available in three different workflow languages—CWL, WDL, and Nextflow—and can be run in diverse cloud platforms, including widely used platforms crucial to this era of collaborative genomics research. We show that rMATS-cloud processes a large pediatric AML dataset of 1113 BAM files in less than 6 h for less than $40. While the HPC version of rMATS-turbo itself is a fast and versatile software, rMATS-cloud provides the same robust performance for datasets which are stored on the cloud without requiring the data to be downloaded. rMATS-cloud saves space and time, by allowing data to be analyzed where it is stored. This broadens accessibility to a global audience, which is increasingly leveraging the cloud to analyze genomics data in a highly collaborative environment.

While the rMATS-turbo software does not directly operate on single-cell or spatial RNA-seq data, it has been adapted for these applications [11,12]. Therefore, with additional customization, we envision that rMATS-cloud can be adapted for cloud-based alternative splicing analysis of single-cell and spatial RNA-seq datasets.

Some additional considerations when working with biomedical data in a cloud environment include managing privacy and security. For researchers looking to house large-scale data, many platforms such as the Cancer Genomics Cloud [8] allow users to perform genomic analysis in workspaces that can be kept private or shared with collaborators. Although the responsibility of proper data stewardship and governance is up to users, many platforms provide consistent and secure frameworks for securing data where it is stored.

rMATS-cloud expands the repertoire of available cloud analysis tools for RNA-seq data, enhancing data sharing and reuse by better supporting bioinformatics analysis on collaborative platforms. Each cloud platform has a graphical user interface that allows users to customize workflow configuration settings and manage their large-scale data without extensive computational expertise. rMATS-cloud workflows are available to download from Dockstore and GitHub (https://github.com/Xinglab/rmats-turbo/).

## Code availability

rMATS-cloud is available at https://dockstore.org/workflows/github.com/Xinglab/rmats-turbo/rmats-turbo-cwl, https://dockstore.org/workflows/github.com/Xinglab/rmats-turbo/rmats-turbo-wdl, and https://dockstore.org/workflows/github.com/Xinglab/rmats-turbo/rmats-turbo-nextflow.

## CRediT author statement

**Jenea I. Adams:** Conceptualization, Methodology, Resources, Formal analysis, Writing – original draft. **Eric Kutschera:** Methodology, Resources, Formal analysis. **Qiang Hu:** Methodology, Resources. **Chun-Jie Liu:** Methodology, Resources, Formal analysis. **Qian Liu:** Methodology, Resources. **Kathryn Kadash-Edmondson:** Writing – original draft. **Song Liu:** Conceptualization. **Yi Xing:** Conceptualization, Methodology, Formal analysis, Writing – original draft. All authors have read and approved the final manuscript.
